# Teaching an Old Drug a New Trick: Targeting Treatment Resistance in Genitourinary Cancers

**DOI:** 10.33696/signaling.5.112

**Published:** 2024

**Authors:** Karina Aguilar, Anuj K. Sharma, Tianyu Yang, Dipen Mehta, Chandramukhi S. Panda, Vinata B. Lokeshwar

**Affiliations:** 1 Department of Biochemistry and Molecular Biology, Medical College of Georgia, Augusta University, Augusta, GA, 1410 Laney Walker Blvd., 30912, USA

**Keywords:** HAS3, Hyaluronic acid, UGT-1A9, Hymecromone/4-methylumbelliferone, Genitourinary cancer, Sorafenib

## Abstract

In the quest for improving the clinical outcome of patients with metastatic genitourinary cancers, including metastatic renal cell carcinoma (mRCC), the emphasis often is on finding new targeted therapies. However, two studies by Jordan *et al. (Oncogenesis 2020)* and Wang *et al. (Cancer Cell Int 2022)* demonstrate the feasibility of improving the efficacy of a modestly effective drug Sorafenib against mRCC by attacking a mechanism hijacked by RCC cells for inactivating Sorafenib. The studies also identified hyaluronic acid synthase -3 (HAS3) as a bonafide target of Sorafenib in RCC cells. The studies demonstrate that an over-the-counter drug Hymecromone (4-methylumbelliferone) blocks inactivation of Sorafenib in RCC cells and improves its efficacy against mRCC through the inhibition of HAS3 expression and HA signaling. In the broader context, improving the efficacy of “old and failed drugs” that have favorable safety profiles should increase the availability of effective treatments for patients with advanced cancers.

## Introduction

An armamentarium of drugs that target different molecular pathways, and immunotherapy, have been approved for disseminated/metastatic renal cell carcinoma (RCC) either as single agents or as combination treatments [1-3]. However, poor prognosis despite treatment and treatment related side effects demands a constant quest for finding newer drugs that target previously unchallenged molecular targets and pathways in metastatic renal cell carcinoma (mRCC). Two articles by Jordan *et al.* and Wang *et al.*, both from the Lokeshwar group, offer a new perspective of looking at the failed drugs and making them effective – “teaching an old drug a new trick to become effective” [4,5]. The articles provide clinical evidence for a unique pathway hijacked by RCC cells to inactivate Sorafenib, and thereby making it ineffective as an anti-mRCC drug. Moreover, using preclinical models, the articles show that Sorafenib’s combination with an over-the-counter drug 4-methyumbelliferone (Hymecromone) at the human equivalent dose effectively eliminates mRCC without toxicity at the pharmacological dose of Sorafenib. The first article by Jordan *et al.* demonstrates that the levels of UDP-glucuronosyltransferase-1A9 (UGT-1A9) which specifically inactivates Sorafenib are highly elevated in mRCC [4]. In the article by Wang *et al.*, the authors provide evidence for Hyaluronan synthase 3 (HAS3) as the previously unknown but major target of Sorafenib in RCC cells and demonstrate that HAS3 is highly expressed in mRCC [5]. Both studies together show that at the pharmacologically achievable dose, Hymecromone decreases UGT1-A9 expression in RCC cells, thereby allowing Sorafenib to remain active i.e., to decrease HAS3 expression. Moreover, while each drug alone is ineffective at its pharmacological dose, the combination effectively eliminates metastasis, thus making an old, failed drug like Sorafenib effective again. Previous works by this group and others showed that HAS3 and related molecules are highly elevated in genitourinary tumors and promote tumor growth, angiogenesis, metastasis, and treatment-induced resistance [6-22]. Therefore, the mechanisms and the targets identified in the studies by Jordan *et al.* and Wang *et al.* should likely have a broad clinical implication.

The majority of kidney tumors are RCC, among which clear cell RCC is the major histologic subtype while papillary, chromophobe, and tumor in the collecting duct are remaining subtypes; some of the tumors can have a sarcomatoid variant [23]. While the standard treatment for clinically localized RCC is nephrectomy with lymph node dissection, about 1/4^th^ of the newly diagnosed patients have metastatic disease and about 1/4^th^ more develop metastatic disease following radical nephrectomy requiring a systemic treatment approach [24,25]. The prognosis for patients with metastatic renal cell carcinoma (mRCC) remains poor. The survival of patients with mRCC is about 20% survival at two years and less than 10% at five years. Unlike other solid tumors and hematological cancers, RCC is not amenable to chemotherapy or radiation therapy due to treatment resistance [24,26]. Awakening an immunological response with generalized cytokine immune therapy (i.e., interferon-alpha, and interleukin-2 treatment) was a first-line treatment for mRCC up until the mid-2000s. However, poor response rates with these treatments favored the use of anti-angiogenic drugs. RCC is a highly angiogenic tumor with increased production of vascular endothelial growth factor (VEGF) and signaling in the tumor microenvironment [27].

Anti-angiogenic therapy improved the progression-free survival rates with lesser toxicities than the generalized cytokine immune therapy leading to the approval of tyrosine kinase inhibitors (TKIs) such as Sorafenib, Sunitinib, pazopanib, and cabozantinib, axitinib, mTOR inhibitors such as temsirolimus and everolimus and an anti-VEGF monoclonal antibody (bevacizumab) for the treatment of mRCC [3,24,28]. Immunotherapy has been approved for mRCC since 2015. Currently, two drugs Pembrolizumab and Nivolumab that block the immunosuppressive PD-1/PD-L1 axis are approved for the treatment of mRCC. Immunotherapy is often combined with an anti-angiogenic inhibitor (tyrosine kinase inhibitor) because VEGF is known to induce an immunosuppressive tumor microenvironment [3,29]. However, despite the approval of several TKIs, mTOR inhibitors, an anti-VEGF antibody and immunotherapy, mRCC still poses a grim prognosis for patients with dismal overall and relapse-free survival. The challenges for these treatments are therapy resistance and treatment-related side effects [3,24]. A rarely explored avenue to overcome treatment failure is to target the mechanism by which a specific drug is inactivated in tumors. The clinical translation of this approach should be rapid as the pharmacology of FDA-approved drugs is most often well-characterized. Furthermore, if an over-the-counter drug can overcome the mechanism that inactivates an FDA-approved drug, then such a combination in principle can achieve increased efficacy while maintaining a favorable safety profile. The combination of Hymecromone and Sorafenib described in the studies by Jordan *et al.* and Wang *et al.* demonstrate the feasibility of such an approach [4,5].

Sorafenib was the first oral TKI (400 mg twice daily) that was approved for the treatment of advanced RCC in the mid-2000s. Although tumor cells do not express the known targets of Sorafenib such as, PDGF-receptor, c-Kit, VEGF-receptor, a variety of molecular pathways are suggested as Sorafenib targets. However, the activity of Sorafenib against RCC cells is tested at doses higher than the pharmacological dose (~ 5-μM) and few studies have provided both the clinical and preclinical evidence of these targets in mRCC [30,31]. Sorafenib was also found to be inferior to other TKIs, such as Sunitinib, Pazopanib and to have higher frequency of treatment discontinuation in head-to-head comparisons in multi-center clinical trials [32]. Sorafenib is primarily metabolized in the liver by the classical Phase I oxidative mechanism via CYP3A4, followed by the Phase II metabolism to generate water soluble glucuronide metabolites which are excreted. In a minor pathway, Sorafenib is directly metabolized by UGT-1A9 to Sorafenib-glucuronide which is the terminal inactivating transformation [33-35]. Jordan *et al.* showed that the expression of UGT-1A9 is significantly elevated in RCC cells and in patients’ tumors compared to the normal kidney epithelial cells, and the adjacent normal kidney tissues, respectively. Moreover, the levels were an independent predictor of metastasis and overall survival in patient cohorts [4]. The study further demonstrated that UGT-1A9 is expressed in the microsomes of RCC cells and causes inactivation of Sorafenib *in situ*. This is one of the few examples that show tumor cells hijack a minor drug metabolism pathway to inactivate an antitumor drug. With UGT-1A9 being highly elevated in metastatic tumors, it is unsurprising that Sorafenib has low efficacy. The study then relied on a previous observation from the same group that combination of Hymecromone with Sorafenib effectively stopped growth in preclinical RCC models [10]. That study showed that the combination inhibited hyaluronic acid (HA) synthesis and the addition of HA reversed the antitumor effects of the combination. Hymecromone is a choleretic drug for treating biliary aliments and is available for use through European pharmacies. Clinical trials in the US and Europe demonstrate an excellent safety profile for oral Hymecromone doses 300 – 1200 mg three times per day [36-39]. The 50% toxic dose for 4-MU in rodents is 2.8 g to >10 g per kg (Pubchem; CID 5280567). Notably, Hymecromone is the most well-characterized inhibitor of HA synthesis [39-41].

HA is a large polymer non-sulfated glycosaminoglycan consisting of disaccharides D-glucuronic acid and N-acetyl-D-glucosamine. It can be synthesized by one of the three HA-synthases (HAS) – 1, 2, or 3. It is well-established that HA promotes tumor growth, metastasis, and angiogenesis by inducing intracellular signaling by binding to its receptors CD44 and RHAMM [42-45]. Additionally, HA forms a coat around tumor cells, and therefore, protects tumor cells from exposure to cytotoxic agents [4,8,9,13,46,47]. Hymecromone is a competitive inhibitor of HA synthesis with an IC_50_ of 0.4 mM. At this concentration, Hymecromone inhibits tumor cell proliferation, chemotactic motility, invasion, and HA signaling *in vitro* [6,9,13]. It alone is effective in inhibiting tumor growth, metastasis, and angiogenesis in a variety of tumors [48-56]. Lokeshwar’s group has previously shown that at human equivalent dose, Hymecromone inhibits bladder and prostate tumor growth. Furthermore, Hymecromone inhibits both soft tissue and skeletal metastasis in transgenic and experimental metastasis models of prostate cancer without observable serum or tissue toxicity, anti-coagulant or anti-spermicidal activity, or changes in animal weight [6,9,13]. Intriguingly, three studies published by Lokeshwar’s group showed that Hymecromone synergizes with Sorafenib to inhibit RCC growth and metastasis in an orthotopic model at doses lower than the IC_50_ for the inhibition of HA synthesis. Furthermore, at lower doses Hymecromone alone does not inhibit HA synthesis, but rather it inhibits the expression of UGT-1A9 [4,5,10]. This begs the question, how then the combination of Sorafenib with low dose Hymecromone inhibits HA synthesis. The article by Wang *et al.* answers this question. That study demonstrated that Sorafenib alone at concentrations higher than the pharmacological dose inhibited HA synthesis in RCC cells by downregulating HAS3 expression. However, when combined with Hymecromone, the pharmacological dose of Sorafenib could inhibit HAS3 expression, HA synthesis and HA-related signaling that promotes RCC growth and metastasis. The study further showed that HAS3 levels are not only elevated in tumor tissues compared to the adjacent normal kidney tissues, but the levels potentially are independent prognostic indicators of metastasis and patient survival [5].

**Figure 1.**
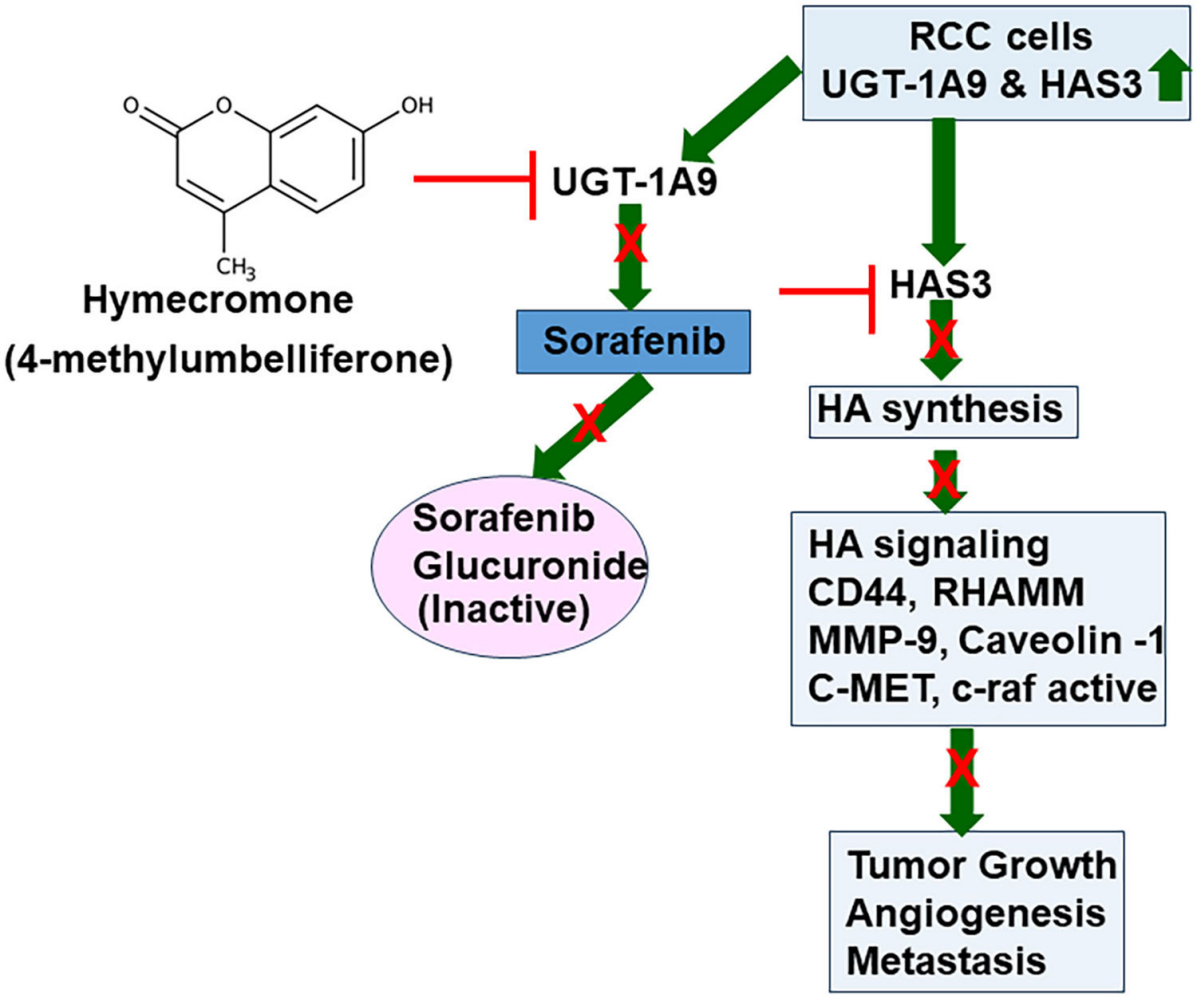
A schematic representation of how Hymecromone improves the efficacy of Sorafenib. UGT-1A9 and HAS3 levels are elevated in RCC tumors. Elevated HAS3 expression increases HA synthesis. HA secreted in the tumor microenvironment binds its cell surface receptors, CD44 and RHAMM on RCC cells and induces intracellular HA signaling that promotes RCC cell proliferation, migration, and invasive activity. On the endothelial cells, HA and HA receptor interaction promotes angiogenic phenotype. Therefore, HAS3 overexpression promotes RCC growth, angiogenesis, and metastasis. Sorafenib downregulates HAS3 expression, however, it fails as an anti-RCC drugs because in RCC cells they express high levels of UGT1A9. Sorafenib is glucuronidated by UGT-1A9 which is an inactive metabolite. By downregulating UGT-1A9, Hymecromone inhibits Sorafenib inactivation in RCC cells, which in turn, inhibits HAS3 expression and HA signaling. Therefore, combination of Sorafenib with Hymecromone both at pharmacological doses effectively inhibits RCC growth and metastasis.

The studies by Jordan *et al.* and Wang *et al.* together support the hypothesis that Sorafenib likely has excellent activity to control mRCC, however, since Sorafenib gets inactivated by UGT-1A9, increased UGT-1A9 levels in mRCC reduce its efficacy, if not making it ineffective. HAS3 and consequently, HA which promote tumor growth and metastasis, is the major target of Sorafenib in mRCC. Since HAS3 and HA levels are elevated in mRCC, Sorafenib should have excellent anti-mRCC activity, provided its inactivation by RCC cells is prevented. The combination of Sorafenib with Hymecromone, which inhibits UGT-1A9 expression, fulfills this requirement. Therefore, the combination demonstrates excellent efficacy against mRCC in preclinical models at the approved dose of Sorafenib (Figure 1). Both studies provide proof for this hypothesis by demonstrating that RCC cells are resistant to the combination of Hymecromone and Sorafenib when UGT-1A9 or HAS3 is ectopically expressed in RCC cells under a viral promoter. Contrarily, knockdown of UGT-1A9 or HAS3 sensitized the cells to Sorafenib alone at the pharmacological dose or lower.

HA is an important component of the extracellular matrix, especially in the cartilage. Therefore, one could envisage a possibility of arthritic pain due to the use of an HA synthesis inhibitor such as Hymecromone. In normal tissues including the cartilage, basal HA levels are low. This HA is of high molecular mass HA (MW ≥ 2x10^6^ Da) and since is deeply embedded in the matrix, it turns over slowly. As in cancer, in inflammatory conditions such as lung fibrosis, acute respiratory distress syndrome (ARDS), HA levels are also elevated which then induces HA-mediated inflammatory signaling. Therefore, the consensus is to reduce high levels of HA in both cancer and inflammatory diseases [8,57,58]. As with any single drug or a drug combination that shows promise in pre-clinical studies, clinical trials are necessary to determine the efficacy and toxicity of the Sorafenib and Hymecromone combination as a treatment for advanced RCC. In preclinical studies, the combination did not affect the basal HA levels and did not have tissue, or serum toxicity and did not affect animal weight [4,5,10]. Given that both Hymecromone and Sorafenib are in human use, these preclinical studies should form a reasonable basis for testing the combination in well-designed clinical trials.

These studies highlight how targeting specific mechanisms that cause the failure of FDA-approved drugs could improve treatment response in patients with cancer while maintaining a drug’s safety profile. Such combinations could be rapidly translated in the clinic since the drug is FDA-approved. Given that the prognosis of patients with mRCC has improved very little over the years despite the approval of several therapeutic agents and immunotherapy being FDA-approved, approaches to improve the efficacy of modestly effective drugs by overcoming their inactivation in mRCC merit further investigation.
